# Development of a Digital Image Processing- and Machine Learning-Based Approach to Predict the Morphology and Thermal Properties of Polyurethane Foams

**DOI:** 10.3390/polym17070928

**Published:** 2025-03-29

**Authors:** Caglar Celik Bayar

**Affiliations:** Department of Metallurgical and Materials Engineering, Zonguldak Bulent Ecevit University, 67100 Zonguldak, Türkiye; caglarbayar@beun.edu.tr or caglarbayar@gmail.com

**Keywords:** polyurethane foam, cyclohexane blowing agent, digital image processing, machine learning, Voronoi tessellation diagram, MP2

## Abstract

Polyurethane foams are frequently used to provide thermal insulation. Thanks to the blowing agents used during their synthesis, pores are created in the structure and thermal insulation is achieved through these pores. In this study, five different insulating polyurethane foam samples containing water and cyclohexane blowing agents were synthesized. Pore stabilities and their effects on pore neighboring were analyzed computationally (MP2/aug-cc-pVDZ). A digital image processing- and machine learning-based algorithm was developed to predict the mean neighboring effect distances of the produced foams. It was created using the Voronoi tessellation method used for the identification problems in industrial applications. This method showed that there was a close relationship between the calculated Voronoi neighboring effect distances of the samples and their thermal conductivity coefficients. Considering the Voronoi neighboring effect distances proposed in this study, the thermal conductivity coefficient of similar polyurethane foams could be predicted. This method required only a standard mobile phone to capture images of the samples and the algorithm developed using Python (version 3.13.2) programming language. In addition, when compared to the local surface imaging device SEM, it allowed the entire surface to be analyzed faster and at once, without any surface deterioration.

## 1. Introduction

Polymeric foams, lightweight materials with easily tunable properties, are the first choice for a wide range of applications, such as packaging, automotive, electronics, furnishing, footwear, aerospace, toys, food contact, or construction materials. Polyurethane (PU) foams, one of the most frequently used types, are generally used in comfort applications or heat and sound insulation materials [1,2,3].

PU foams are synthesized from the reaction between polyols and polyisocyanates using physical and/or chemical blowing agents. Catalysts, surfactants, and fire retardants can be used as additives during the synthesis [4]. The −NHCOO− urethane linkages form the PU structure as presented in Equation (1) [1]. This is an exothermic process [5,6,7].

RNCO (isocyanate) + R’OH (alcohol) → RNHCOOR’ (urethane) + Heat(1)

Foaming is caused chemically or physically or by a combination of the two. The gas produced from the blowing agents fills the cellular structure of the bubbles [8]. Water is generally used as a chemical blowing agent in PU production because it is easily available, harmless, and cheap. It reacts exothermically with isocyanate, producing amine and blowing CO_2_ gas, as shown in Equations (2) and (3) [7,9].

RNCO (isocyanate) + H_2_O → RNHCOOH (carbamic acid) (2)

RNHCOOH → RNH_2_ (amine) + CO_2_ + Heat (3)

Physical blowing agents are also widely used in PU formation. These substances are inert and low-boiling-point liquids that evaporate in situ with increasing heat and temperature in the PU formation reaction. They can provide additional gas during foaming, add some desired properties to the foam, and have the advantage of not consuming isocyanate. The most commonly used physical blowing agents are methyl formate, n-pentane, isopentane, cyclopentane, n-hexane, and cyclohexane [8]. A mixture of chemical and physical blowing agents can be used to provide advantages over either type of blowing agent alone [8,9].

Theoretical foam modeling studies have been the focus of interest of scientists for many years. A theoretical model that showed good agreement with the experimental data was developed to predict the variation in density and temperature with time in rigid PU foams [10]. In the study of Tesser et al. [11], an extended Flory–Huggins model was successfully used to describe blowing agent vapor–liquid equilibrium in reacting mixtures. A series of over 30 differential equations were solved by Al-Moameri et al. [8] to model experimental data on PU foam formation. The simulation results for foam height were consistent with the experimental data. Chemical and physical blowing agent performance limits and their mixture at different blowing agent loadings were simulated with the Matlab code developed for PU foam reactions [9]. It was observed that physical blowing agents formed higher PU foam heights when mixed with water.

Although the most commonly used imaging method on PU foams is SEM, image processing studies have attracted attention in recent years. X-ray microtomography was used to study in situ the uniaxial tensile response of low-density PU foam [12]. A time-resolved X-ray radiography imaging method was used to monitor in situ the foaming process of reactive closed-cell rigid PU foam systems filled with nanofillers [13]. A digital image processing method was used to estimate the foam characteristics of PU in another study [7].

Machine learning algorithms have also been used for polymeric foam analyses in the literature. Machine learning investigations of polylactic acid bead foam extrusion have been performed [14,15]. The prediction task for magnetostriction magnetorheological foam was accomplished using this method as well [16].

The open source Voronoi tessellation diagram method, which was included in PU foam analysis for the first time in this study, was adapted to different solution procedures in the literature. The problems ranged from engineering to science and from computer graphics to game creation. Since the modeling, implementation, and adaptation of solution procedures of this method do not require complex infrastructures, the problems encountered in these areas can be solved easily and accurately. General elastoplastic deformations were analyzed using a Voronoi diagram-based algorithm in the study of Gao et al. [17]. A Voronoi diagram-based algorithm was constructed to position the dosimeters in the desired planes of a product box [18]. In the study of Asakawa et al. [19], Voronoi diagrams were used to create an indicator for stiffener arrangement in stiffened composite panels. Becedas et al. [20] determined the polyhedral norms using Voronoi diagrams and presented a theoretical solution procedure. A new design procedure for variable-porosity porous structures was presented in the study of Chao et al. [21]. The solution method was constructed using a stress line and a Voronoi diagram. The porosities and the stress distributions were analyzed by a 3D Voronoi diagram-based algorithm. Shuai et al. [22] used Voronoi diagrams and polygons to propose a charging demand clustering system for commercial electric vehicles. Chen et al. [23] used Voronoi diagrams to develop a model for partitioning the geographical space constrained by public transport networks. A developed solution procedure using Voronoi cells and diagrams was proposed for the problems related to particle hydrodynamics and particle distribution [24]. The technique developed presented strong and acceptable results for the problem case study that was about violent flows in coastal engineering. A branching method based on Voronoi diagrams was proposed by Chen and Zhu in order to obtain mesh blending tubular surfaces [25]. The aim was to obtain a solution procedure for the problem of varying radius and arbitrary orientation of the branching surface of the tubes. In another study, a 3D Voronoi diagram-based form error estimation method for the fast and accurate inspection of free-form surfaces was proposed [26].

### The Aim of This Study

The aim of this study can be expressed as follows: Cyclohexane (CH) blowing agent alone is insufficient to create foam because it has a high boiling point, a high heat of vaporization, and high viscosity. Advantageously, it is an environmentally friendly chemical as it has zero ODP (ozone depletion potential) and GWP (global warming potential) values [8]. In this study, CH and water were used together to ensure easier evaporation of CH and increase its contribution to foam formation (Note that CH evaporates easier using the heat and temperature increase in the PU and CO_2_ formation reactions presented above). For this purpose, five different PU samples containing a combination of CH and water blowing agents were prepared. In the experiments, the amount of water was kept low and constant; however, the values of CH wt.% were changed increasingly to observe the effect of this agent on foam formation. The resulting PU foam images were captured with a 50 MP standard mobile phone camera, and the mean neighboring effect distances between the pores of each sample were calculated using the open source Voronoi tessellation method. Afterwards, the thermal conductivity coefficient of each sample was measured instrumentally. It was investigated whether there was a relationship between these two values. If so, this could be a good example for a machine learning algorithm where the thermal conductivity coefficient of an unknown sample with the same chemical content could be predicted simply by calculating the mean Voronoi neighboring effect distance in its captured image. It was concluded that such a relationship indeed existed, and the proposed machine learning algorithm could be used for similar PU foams.

## 2. Materials and Methods

### 2.1. Materials

Commercially available PU casting components, component A (polyol) and component B (PMDI: polymethylene polyphenyl isocyanate or polymeric MDI), were obtained from Verpol (İstanbul, Türkiye) for the synthesis of insulating PU foams [27]. The physical properties of polyol were given as 120 mg KOH g^−1^ hydroxyl value, 0.97 g cm^−3^ density, and 130 mPa s viscosity, while those of PMDI were given as 31.2 NCO%, 1.12 g cm^−3^ density, and 195 mPa s viscosity. The 1:1 (*v*/*v*) mixture of these two components has generally been used in home repairs under the name of hard plastic. However, this study aimed to synthesize insulating PU foams by adding the mixture of water as a chemical blowing agent and CH as a physical blowing agent. Distilled water and CH with a purity of 99.77% and a density of 0.782 g ml^−1^ were used in the experiments. The combination of the CH molecule, which physically expanded as the ambient temperature increased during the exothermic PU formation and bubbling reaction [5,6,7,9], and the CO_2_ molecule obtained as a result of the reaction between PMDI and water [7,9] enabled the formation of air bubbles that gave the insulation feature to the PU samples. The mass of polyol, PMDI, and water in the samples was kept constant; only the mass of CH was varied to observe its effect on material insulation. Thus, five different samples with CH wt.% values ranging from 0.4 to 10 were prepared (Table 1). The sample containing only water blowing agent was considered as the reference sample. Since CH alone was not enough to provide foaming, it was observed that no bubbles formed in the mixtures containing only CH and no water.

In sample preparation, first, water, CH, and polyol were mixed in a cardboard cup, and then PMDI was poured onto them slowly. Then, all the components were mixed homogeneously again, and after a while, CO_2_ was released exothermically. Simultaneously, with the increase in ambient temperature, the CH molecules physically expanded, contributing to bubble formation. The obtained closed-shell PU foam samples cooled and solidified after a while, and the thermal conductivity coefficients were measured with the KD2 Pro Thermal Properties Analyzer, which is battery-operated and has a special probe [28]. SEM images of the samples were taken with a high-resolution FEI QUANTA FEG 450 instrument. Gold nanocoating was applied to the samples to create conductivity. High vacuum at 6.00 and 8.00 kV was used in the analyses.

### 2.2. Computational Chemistry Methods

The quantum chemical modeling used in this study was based on the following principles: Since CH is a viscous liquid, as the amount of CH added to the reaction medium increased, the ambient pressure was also assumed to increase. As a result, the volume occupied by the CO_2_ gas and the accompanying CH gas was assumed to decrease. Since the amount of water (chemical blowing agent) was constant and the produced CO_2_ gas was limited in the experiments, the amount of CH (physical blowing agent) was thought to be very decisive in thermal conductivity coefficient values. For this reason, CH was considered a viscous liquid solvent, and the thermodynamic stability of the evaporated CH gas in this solvent was calculated computationally at different conditions. It was worth noting that the experimental ambient temperature was reported to increase to approximately 393.15 K (120 °C) when water and CH blowing agents were used together [9]. Accordingly, starting from normal conditions, the temperature was increased to 393.15 K and the pressure was increased to 150 atm in the calculations. The IEFPCM SCRF method existing in the Gaussian 09 program software was used for solvent modeling [29]. Standard formation Gibbs free energies of CH gas under different conditions were calculated at the MP2/aug-cc-pVDZ theoretical level [30]. The energies of all the minima considered on the potential energy surface included zero-point energy corrections and had no imaginary frequencies, indicating that they were neither transition states nor saddle points.

### 2.3. Voronoi Tessellation Method

Voronoi tessellation can be considered a highly proper methodology for foam morphology characterization because this method has ability to represent the complex and irregular structures commonly found in foam systems. Foam morphology involves typically understanding and integration of the distribution, shape, and connectivity of pores within the foam, which requires capturing both geometric and spatial features. Voronoi tessellation is also suitable for modeling and analyzing thermal properties in foams due to several key reasons related to the way it represents spatial structure and the properties of the foam. A couple of detailed breakdowns of why Voronoi tessellation is a suitable method for exploring thermal properties can be listed as follows: The Voronoi tessellation method captures the heterogeneous and irregular geometry of foam cells, crucial for modeling how heat is conducted through the foam. It also has the ability to represent the cell structure accurately (e.g., walls, faces, edges), which directly influences heat transfer. Foams are often composed of irregularly shaped cells with walls and pores, and their morphology (structure) is highly heterogeneous. Voronoi tessellation provides a natural way of representing this type of structure because it divides space into regions based on the neighboring effect distances of pores.

Many image analysis methods (such as pixel-based analysis or traditional image segmentation) using readily available software could likely be developed for performing a thermal analysis and predicting the morphology and thermal properties of foams. What distinguishes Voronoi tessellation from such traditional image analysis methods is that Voronoi tessellation simplifies the representation of the foam structure. Instead of processing every individual pixel in an image, Voronoi tessellation provides a compact mathematical representation of the foam’s morphology. This reduces the computational load. Furthermore, Voronoi tessellation can adapt to varying resolutions and cell sizes, which allows for the flexible modeling of different scales of foam structure; this enables the use of standard images taken via a standard mobile phone camera. Notably, a 50 MP standard phone camera was used in this study since many people have this type of mobile phone. It is clear that the resolution and quality of the images taken by a camera would be crucial for an accurate Voronoi analysis. Higher-quality cameras provide better results; however, Voronoi tessellation is a strong method that still gives adequate prediction results, even if the quality and resolution of an image are not perfect.

The theory of the Voronoi tessellation method is as follows: Assume that a set of a site is given as P = {p_1_, p_2_,…, p_n_}. A Voronoi tessellation is the subdivision of n-cells in the work space. Each cell is described for each site in P with the characteristic that a point q lies in the cell corresponding to a site of p_i_. The site is created via the property of d(p_i_, q) < d(p_j_, q). In a Voronoi tessellation, each segment corresponds to the points that are in the plane equidistant to the two sites, nearest to each other. This method is commonly used in the field of game theory, machine learning, robotics, mechatronics, artificial intelligence, computer science, chemistry, physics, civil and environmental sciences, etc.

**Figure 1:**
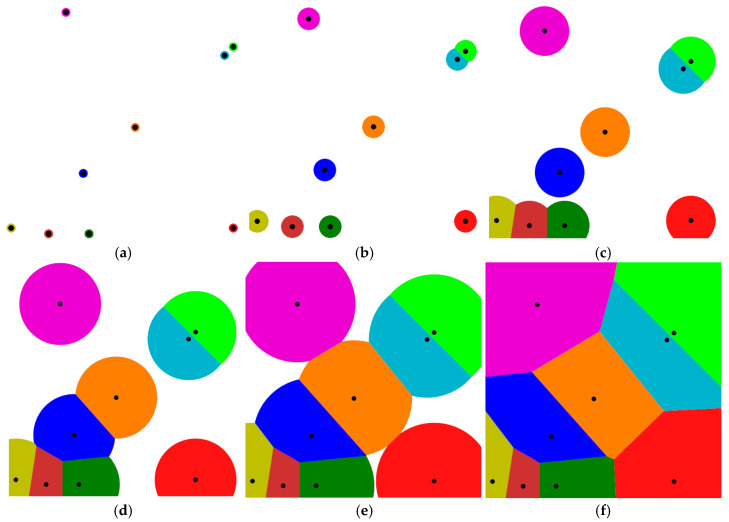
A simple schematic model for creating Voronoi cells is shown in (**a**–**f**). In this model, the data points that are randomly scattered in a plane are used to create a Euclidean metric. The effective area for each point is increased until the surrounding regions collide and be-come unified. Each region forms a cell corresponding to that point. As a result, the combi-nation of data sets forms the underlying space and is called Voronoi tessellation.

A simple schematic model for creating Voronoi cells is shown in Figure 1a–f [31]. In this model, the data points that are randomly scattered in a plane are used to create a Euclidean metric. The effective area for each point is increased until the surrounding regions collide and become unified. Each region forms a cell corresponding to that point. As a result, the combination of data sets forms the underlying space and is called Voronoi tessellation.

**Figure 2:**
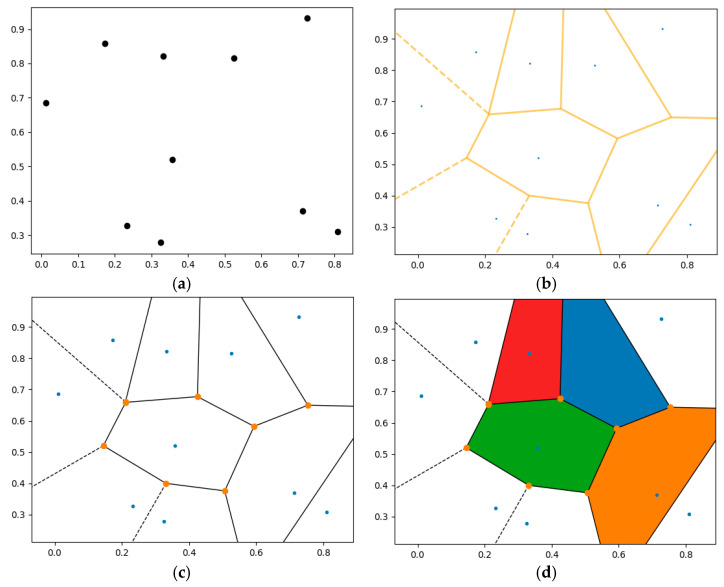
The Voronoi diagram created using the Python algorithm: (**a**) 10 data points used, (**b**) built Voronoi diagram plot, (**c**) indicated Voronoi vertices, and (**d**) colored Voronoi diagram.

The Voronoi tessellation model used in this study was prepared using the Python (version 3.13.2) programming language, supporting an open source strategy [32]. The Voronoi diagram created with this algorithm is shown with an example in Figure 2. Here, 10 data points are used (Figure 2a), and the Voronoi diagram graph is built (Figure 2b). Then, related Voronoi vertices are indicated (Figure 2c), and the diagram is colored (Figure 2d).

### 2.4. Digital Image Processing, Segmentation, Identification, Outlier Handling, and Prediction

**Figure 3:**
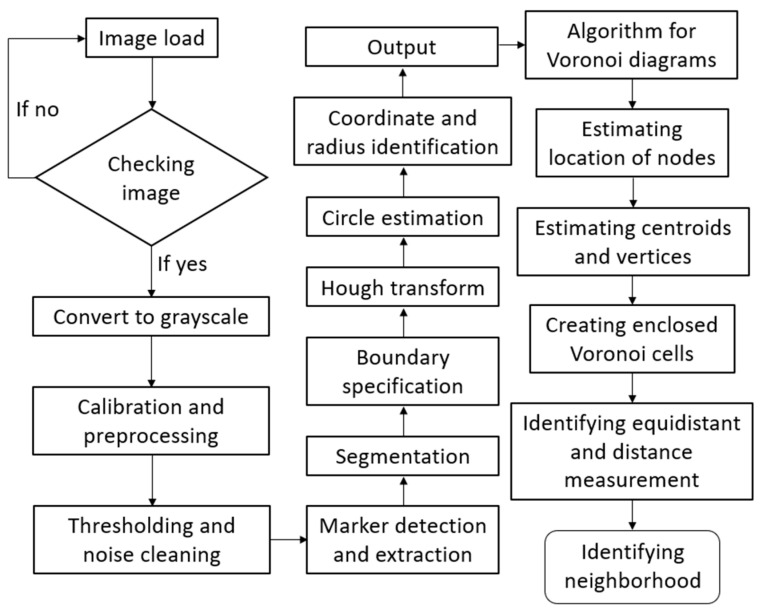
Flow chart showing the details of the Voronoi-based prediction algorithm proposed in this study.

The flow chart showing the details of the proposed Voronoi-based prediction algorithm in Figure 3 can be explained as follows: To use Voronoi tessellation for the prediction of thermal properties in a foam, it is essential to first carry out the digital image processing of the material. The steps of image segmentation, identification of pore centers, and handling of outliers are critical to ensure that the Voronoi tessellation is accurately applied to the material’s structure. The first step is processing the image of the foam material to extract relevant features, such as detecting boundaries of the cells in the foam structure. Image segmentation includes key steps for isolating the individual cells and distinguishing the solid parts from the pores. These steps involve grayscale conversion of the image, thresholding, edge detection, and morphological operations (e.g., dilation, erosion, closing, opening, etc.). After the image is segmented and the foam structure is specified, the pore centers, which are used as Voronoi cells for Voronoi tessellation, are identified. This task is performed by following the Hough transform approach. In the foam structure, it is assumed that the pore geometries are almost circular or nearly circular so that the Hough transform can detect the circular shapes in the image, which are to be identified as the pore centers. During the segmentation and identification processes, outliers can be seen because of some reasons like noise, misclassifications, or image quality. Such outliers may indicate incorrectly specified pores and their centers, which negatively affect the Voronoi tessellation and thermal property prediction processes. They are filtered using a simple size filtering and neighboring (proximity) filtering algorithm. The next step is Voronoi tessellation for the thermal property prediction procedure. This process includes partitioning the foam structure into regions based on the pore centers that are identified. Pore centers are connected to create Voronoi cells, which form Voronoi diagrams. This Voronoi tessellation process creates a set of polygons representing the cells in the foam structure, which enables to predict the neighboring and neighboring effects between the cells.

## 3. Results and Discussion

### 3.1. The Foam Density and Thermal Conductivity Relationship

**Figure 4:**
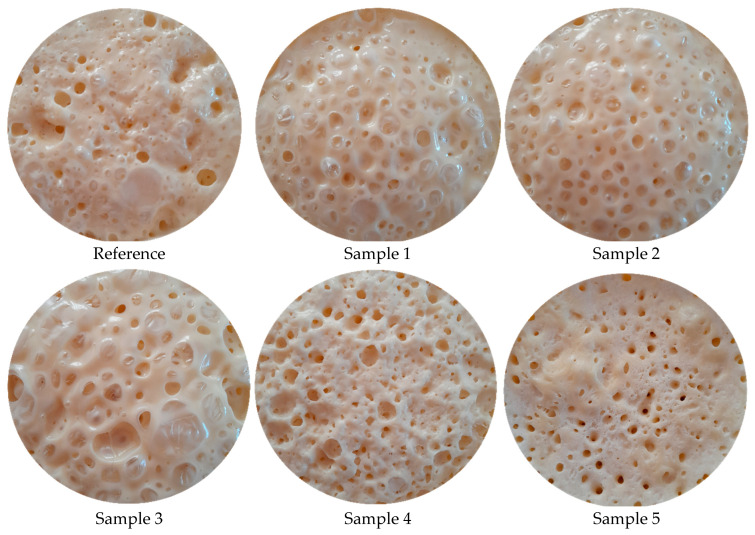
The original images of PU foam samples.

The original images of the PU foam samples are shown in Figure 4. At first glance, the pores in the CH-containing samples are more evident and increased in number compared to the CH-free reference sample. It can be observed that the foam density decreased when low wt.% CH was used in the foams and increased when high wt.% CH was used. This was because CH, a high-viscosity liquid, prevented pore formation by covering the foam surface and increased foam density when used in high amounts (Table 1 and Table 2) (also see SEM images in further sections). Additionally, in Samples 1 to 5, it can be observed that, as the foam densities increased, the thermal conductivity coefficients increased and the insulation weakened (Table 2). At CH wt.% values of 4 and below (Samples 1−3), insulating materials were obtained compared to the reference sample. However, there was no improvement in thermal insulation in Sample 4 (containing 7 wt.% CH), since it had the same thermal conductivity coefficient as the reference sample. When this ratio increased to 10%, it was observed that the conductivity increased even more in an unpreferred manner (Sample 5). The following result was obtained from the measurements: the addition of physical blowing agent CH to the chemical blowing agent of water improved the thermal insulation of the considered PU foam materials up to the level of 4 wt.% CH addition.

### 3.2. Molecular Modeling Results

**Figure 5:**
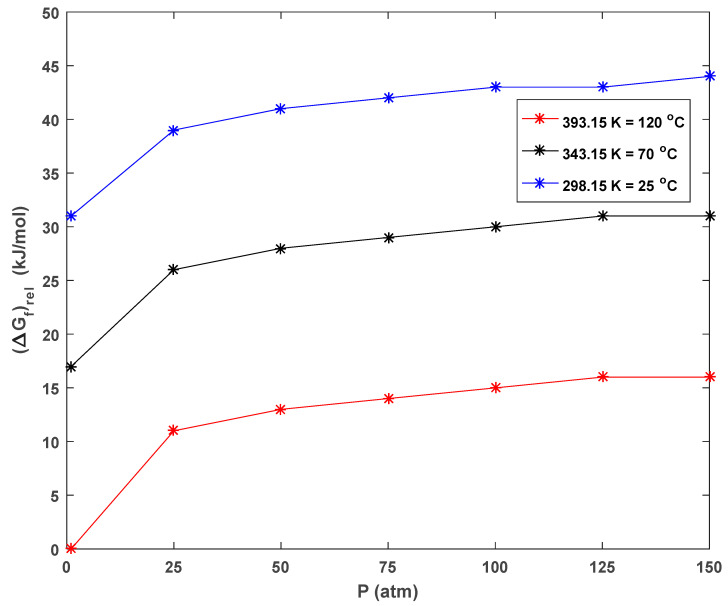
Graphical representation of the relative thermodynamic stability of CH bubbles in CH solvent at various pressures and temperatures.

The calculated Gibbs free energies of the formation (Δ*G_f_*) of CH gas under different conditions in CH solvent using the MP2/aug-cc-pVDZ theoretical level are listed in Table 3. It was concluded that the thermodynamic stability of CH gas was maximum at high temperatures and low pressures, and the stability tended to decrease as the pressure increased at the same temperature. It was thermodynamically most stable at a temperature of 393.15 K (120 °C) and a pressure of 1 atm (Table 3 and Figure 5), which were considered as a reference for the computational results (Δ*G_f_* = 0 kJ mol^−1^). Based on these results, it was possible to make the following comments: CH added to the medium at high wt.% increased the ambient pressure and formed fewer stable gas bubbles. The decrease in the number of stable bubbles resulted in a decrease in neighboring effects and an increase in neighboring effect distances in the foam. These facts were algorithmically calculated and are discussed in detail in the following sections.

### 3.3. Voronoi Tessellation Algorithm Results

The pores in the samples were detected and predicted using the Python programming language-based image processing algorithm. The detection and prediction models were constructed by the Hough transform approach. This approach is commonly used by computer scientists and robotic researchers to detect and predict lines, circles, and curves in real-time applications.

**Figure 6:**
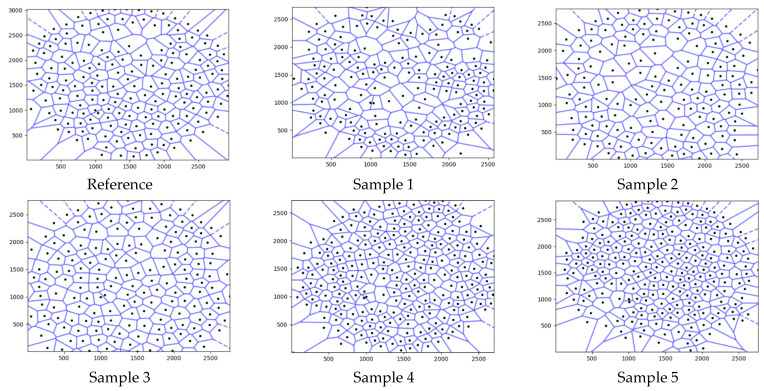
Voronoi tessellation diagrams created using the pore centers.

The Voronoi tessellation diagrams created using the pore centers are shown in Figure 6. As seen in the figure, Voronoi diagrams with different characteristics were formed depending on the pore centers, their location, and the number of pores.

**Figure 7:**
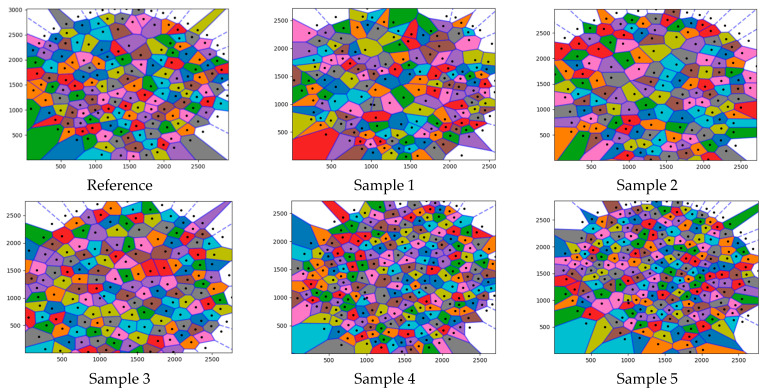
Colored versions of the Voronoi tessellation diagrams in Figure 6.

Colored versions of the Voronoi tessellation diagrams in Figure 6 are presented in Figure 7 to specify the differences. The characteristic of each colored Voronoi diagram contributed to the overall behavior of the diagrams. The colored diagrams were also used to determine each neighboring effect area.

**Figure 8:**
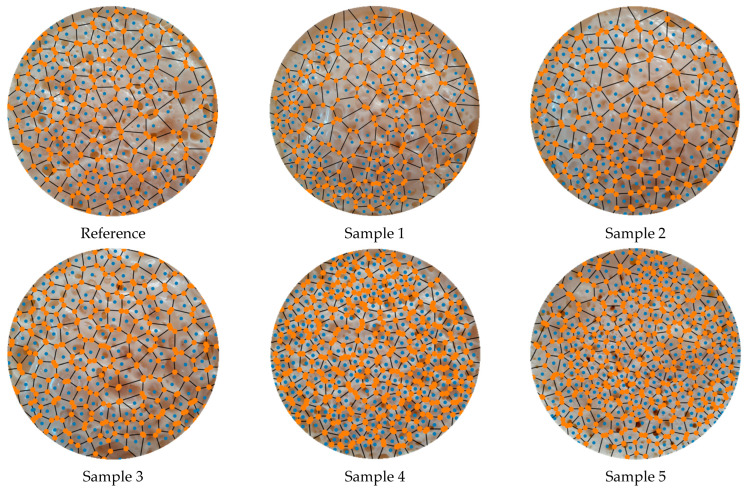
Voronoi vertices and edges of the samples.

**Figure 9:**
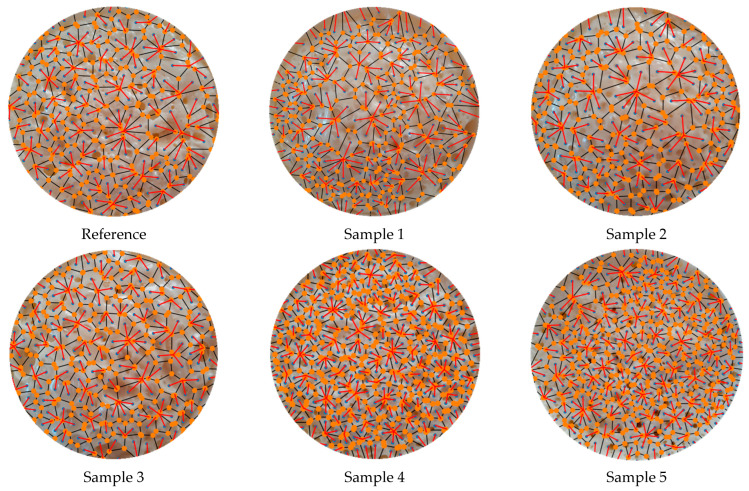
Voronoi tessellation diagrams after detecting the nearby vertices, specifying and removing the outliers, and calculating the distance from each vertex to an initial point.

Voronoi vertices and edges of the samples are demonstrated in Figure 8. They were used to define the nearby vertices, specify and remove the outliers, and calculate the distance of each vertex to an initial point (Figure 9). The effects of neighboring were calculated and visualized in this way.

**Figure 10:**
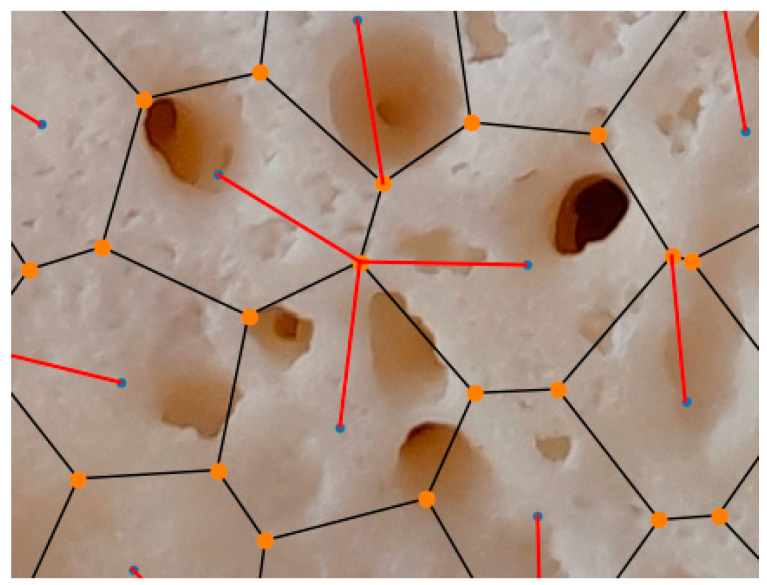
A zoomed-in view of Sample 5 in Figure 9 is presented in Figure 10. It illustrates the details of the detection process of nearby vertices. In this figure, the blue points indicate the effective neighboring points between Voronoi polygons, the black lines represent Voronoi edges, and the orange points are used to denote Voronoi vertices. In addition, the red line shows the distance of effective neighboring from a Voronoi vertex and determines how strong the neighboring effect between closed neighboring regions is.

Note that the pore circles and their centers are not generally shown in Voronoi diagram representations for a clear view. A zoomed-in view of Sample 5 in Figure 9 is presented in Figure 10. It illustrates the details of the detection process of nearby vertices. In this figure, the blue points indicate the effective neighboring points between Voronoi polygons, the black lines represent Voronoi edges, and the orange points are used to denote Voronoi vertices. In addition, the red line shows the distance of effective neighboring from a Voronoi vertex, determining how strong the neighboring effect between closed neighboring regions is.

**Figure 11:**
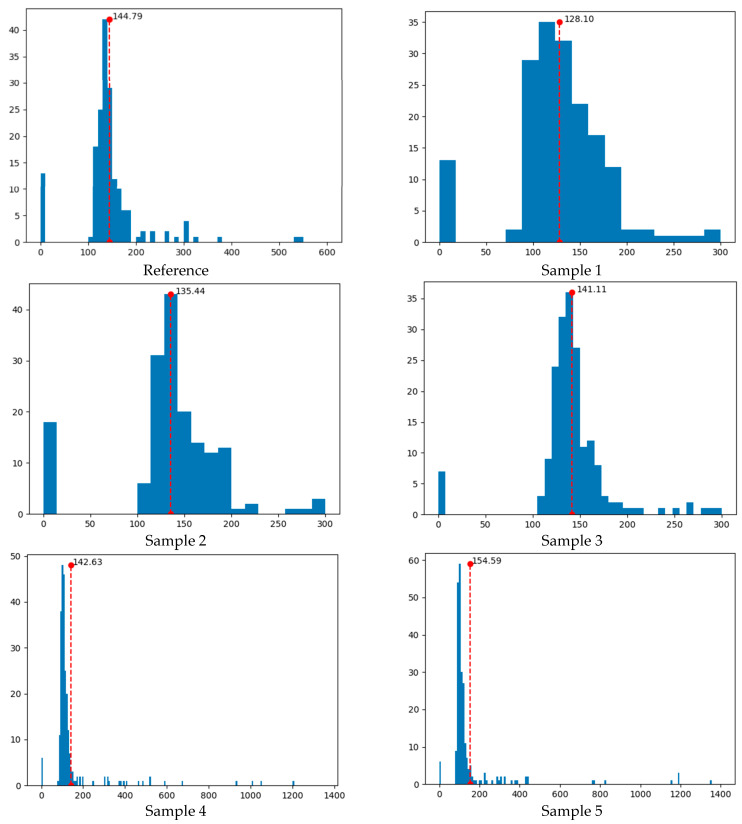
Histogram representation of neighboring effect distances in Figure 9. The *x*-axis represents the neighboring effect distances in pixels, while the *y*-axis represents the frequency of neighboring pores. The red dashed lines show the mean neighboring effect distances. In the studied samples, one pixel is equal to 20.80 µm, which means that the neighboring effect distances range from 2.66 to 3.22 mm.

The histogram representation of the neighboring effect distances in Figure 9 is shown in Figure 11. There, the mean values of the distances are also presented. These values were determined as 144.79 pixels for the reference sample and 128.10, 135.44, 141.11, 142.63, and 154.59 pixels for Samples 1−5, respectively, in increasing order. In the samples examined, one pixel was equal to 20.80 µm, meaning that the neighboring effect distances varied between 2.66 and 3.22 mm. Note that the thermal conductivity coefficients were previously given as 0.093 W m^−1^ K^−1^ for the reference sample and 0.073, 0.074, 0.087, 0.093, and 0.12 W m^−1^ K^−1^ for Samples 1−5, respectively (Table 2). Accordingly, it was concluded that the increase in the mean neighboring effect distances in the samples was parallel to the increase in the measured thermal conductivity coefficients. In other words, as the mean neighboring effect distances increased, thermal insulation decreased in the samples.

Additionally, the proposed Voronoi tessellation-based machine learning algorithm predicted the thermal conductivity coefficients as 0.093 W m^−1^ K^−1^ for the reference sample and 0.066, 0.080, 0.090, 0.093, and 0.12 W m^−1^ K^−1^ for Samples 1−5, respectively. The % relative errors were calculated as 0 for the reference sample, Sample 4, and Sample 5 and 10, 8, and 3 for Samples 1−3, respectively. It was concluded that, as the mean neighboring effect distance in the samples increased, the % relative error decreased and reached 0. This was an expected result because the number of Voronoi cells decreased as pore neighboring in the samples decreased. Statistical performance metrics for the prediction process were obtained as 0.94 for R^2^ (coefficient of determination) and 0.0040 for RMSE (root mean square error) at a 95% confidence interval. The statistical analyses showed that the proposed machine learning algorithm was successful in providing predictions close to experimental values.

It was possible to say that the pores in Samples 1−3 showed a better distribution than the reference sample, i.e., they better represented the normal distribution curve (Figure 11). An important outcome of this situation was that the thermal insulation of these samples was better than the reference sample. The best normal distribution curve belonged to Sample 3, in which 4 wt.% CH had been used. It was observed that the pore distributions were irregular and far from normal distribution compared to the reference sample in Samples 4 and 5, which contained relatively high amounts of CH (7 and 10 wt.%, respectively). Another important observation was that although the thermal conductivity coefficients of the reference sample and Sample 4 were the same (0.093 W m^−1^ K^−1^), the pore distribution histograms were quite different from one another. However, the mean neighboring effect distances were close in both samples, the pores showed a highly asymmetrical distribution towards the left of the mean value in Sample 4. In general, it was concluded that measuring the thermal conductivity coefficient alone was not sufficient to understand the thermal properties of such PU foams, as the Voronoi tessellation histograms contained more detailed information about the distribution and neighboring of pores on the material surface.

### 3.4. SEM Results

**Figure 12:**
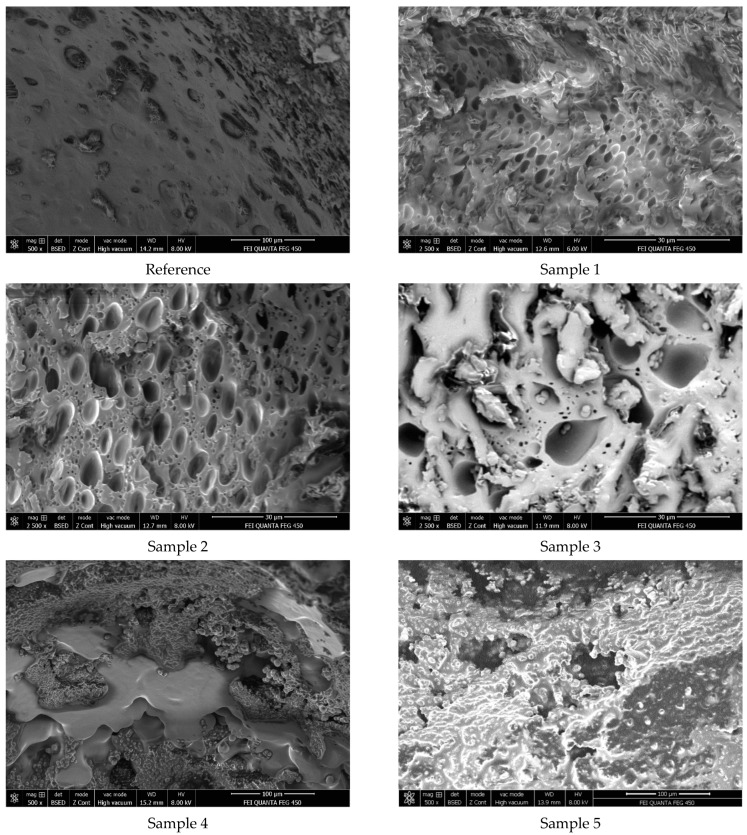
SEM images of PU foam samples.

The mean neighboring effect distances of the pores on the polyurethane sample surfaces were predicted in millimeter scale using a 50 MP standard mobile phone camera. The conclusion from the previous sections was that, as the CH wt.% in the samples increased, the mean neighboring effect distances of the pores also increased. At that point, it was a matter of curiosity whether this trend in neighboring distances would be similar at the micron level. Therefore, SEM analyses of the samples were carried out to instrumentally elucidate and verify this (Figure 12). When the SEM images were examined, it was observed that the increase in CH wt.% in the samples significantly increased the neighboring distances of the pores from Sample 1 to Sample 5, as expected. While the neighboring distances were quite small in Samples 1 and 2, they gradually increased between Samples 3 and 5. With the use of increasing amounts of CH in Samples 3 to 5, the surface became blurry by being covered with high-viscosity CH, and as a result, pore neighboring gradually decreased.

## 4. Conclusions

A close relationship emerged between the calculated mean Voronoi neighboring effect distances and the measured thermal conductivity coefficients of the examined samples. Considering these Voronoi distances of the samples as a reference, it was concluded that the thermal conductivity coefficient of an unknown sample with the same chemical content could be predicted only with the digital image processing- and Voronoi tessellation-based machine learning algorithm proposed in this study. (Note that digital images only give information on the surface morphology of PU foams, while thermal conductivity is a bulk property, and interior structure cannot be ignored. Therefore, it is very important for the validity of the proposed algorithm that the chemical contents of the foams are similar.) What distinguishes this work from the studies existing in the literature is that the methodology presented in this paper is systematic, since it is designed to meet the expectations of the end user. It also looks for a solution which does not need to use special laboratory tools, high-cost sensors, computers, embedded systems, etc. The methodology introduced is comprehensive, since it combines a problem, a modeling structure, a solution procedure, successful experiments, and verifications.

The Voronoi-based machine learning algorithm proposed in the study and SEM analyses gave a similar trend in pore neighboring. This trend was quite significant because it was closely related to the thermal conductivities of the samples. Although surface scans can be performed with SEM analyses, it is known that this method has some disadvantages such as performing local scanning, burning sample surfaces at high voltage values, and using a costly gold plating method to create conductivity in non-conductive samples. Therefore, it was concluded that the proposed algorithmic method, which allows one to scan the entire foam surface at once without any surface deterioration, can perform faster, and is much cheaper, may be used as an alternative to SEM.

## Figures and Tables

**Table 1:** Amounts of chemicals used in the samples.

	Polyol (mL)	Polyol (g)	PMDI (mL)	PMDI (g)	Water (mL)	Water (g)	CH (mL)	CH (g)	CH (wt.%)
Reference	30	29.1	30	33.6	0.3	0.3	0	0	0
Sample 1	30	29.1	30	33.6	0.3	0.3	0.3	0.2	0.4
Sample 2	30	29.1	30	33.6	0.3	0.3	1.5	1.2	2
Sample 3	30	29.1	30	33.6	0.3	0.3	3.0	2.3	4
Sample 4	30	29.1	30	33.6	0.3	0.3	6.0	4.7	7
Sample 5	30	29.1	30	33.6	0.3	0.3	9.0	7.0	10

**Table 2:** The density and thermal conductivity relationship of the samples.

Sample	Density	Thermal Conductivity Coefficient
	(kg m^−3^)	(λ) (W m^−1^ K^−1^)
Reference	633.30	0.093
Sample 1	512.87	0.073
Sample 2	514.62	0.074
Sample 3	547.55	0.087
Sample 4	581.81	0.093
Sample 5	768.16	0.12

**Table 3:** Calculated Gibbs free energies of formation (Δ*G_f_*) of CH gas under different conditions in CH solvent using MP2/aug-cc-pVDZ theoretical level.

***T* = 393.15 K (120 °C)**							
*P* (atm)	1	25	50	75	100	125	150
(Δ*G_f_*) (au)	−234.9921	−234.9881	−234.9873	−234.9868	−234.9864	−234.9861	−234.9859
(Δ*G_f_*)*_rel_* (au)	0	0.004006	0.004869	0.005374	0.005732	0.006010	0.006237
(Δ*G_f_*)*_rel_* (kJ mol^−1^)	0	11	13	14	15	16	16
***T* = 343.15 K (70 °C)**							
*P* (atm)	1	25	50	75	100	125	150
(Δ*G_f_*) (au)	−234.9857	−234.9822	−234.9815	−234.9810	−234.9807	−234.9805	−234.9803
(Δ*G_f_*)*_rel_* (au)	0.006422	0.009920	0.010673	0.011114	0.011426	0.011669	0.011867
(*ΔG_f_)_rel_* (kJ mol^−1^)	17	26	28	29	30	31	31
***T* = 298.15 K (25 °C)**							
*P* (atm)	1	25	50	75	100	125	150
(Δ*G_f_*) (au)	−234.9802	−234.9772	−234.9765	−234.9762	−234.9759	−234.9757	−234.9755
(Δ*G_f_*)*_rel_* (au)	0.011910	0.014949	0.015604	0.015987	0.016258	0.016469	0.016641
(Δ*G_f_*)*_rel_* (kJ mol^−1^)	31	39	41	42	43	43	44

## Data Availability

The raw data supporting the conclusions of this article will be made available by the author on request.
